# Mechanistic insights from a quantitative analysis of pollen tube guidance

**DOI:** 10.1186/1471-2229-10-32

**Published:** 2010-02-22

**Authors:** Shannon F Stewman, Matthew Jones-Rhoades, Prabhakar Bhimalapuram, Martin Tchernookov, Daphne Preuss, Aaron R Dinner

**Affiliations:** 1 Department of Chemistry, The University of Chicago, 929 E 57th St, Chicago, IL 60637, USA; 2 James Franck Institute, The University of Chicago, 929 E 57th St, Chicago, IL 60637, USA; 3 Department of Molecular Genetics and Cell Biology, CLSC 1106, 920 E 58th St, Chicago, IL 60637, USA; 4 Current address: Chromatin, Inc, 3440 S Dearborn St, Suite 280, Chicago, IL 60616, USA; 5 Institute for Biophysical Dynamics, The University of Chicago, 929 E 57th Street, Chicago, IL 60637, USA; 6 Department of Physics, The University of Chicago, 5740 S Ellis Ave, Chicago, IL 60637, USA; 7 Department of Biology, Knox College, 2 E South St, Galesburg, IL 61401-4999, USA; 8 International Institute of Information Technology, Gachibowli, Hyderabad 500 032, Andhra Pradesh, India; 9 Current address: Department of Physiology and Biophysics, Albert Einstein College of Medicine of Yeshiva University, Jack and Pearl Resnick Campus, 1300 Morris Park Ave, Bronx, NY, 10461, USA

## Abstract

**Background:**

Plant biologists have long speculated about the mechanisms that guide pollen tubes to ovules. Although there is now evidence that ovules emit a diffusible attractant, little is known about how this attractant mediates interactions between the pollen tube and the ovules.

**Results:**

We employ a semi-*in vitro *assay, in which ovules dissected from *Arabidopsis thaliana *are arranged around a cut style on artificial medium, to elucidate how ovules release the attractant and how pollen tubes respond to it. Analysis of microscopy images of the semi-*in vitro *system shows that pollen tubes are more attracted to ovules that are incubated on the medium for longer times before pollen tubes emerge from the cut style. The responses of tubes are consistent with their sensing a gradient of an attractant at 100-150 *μ*m, farther than previously reported. Our microscopy images also show that pollen tubes slow their growth near the micropyles of functional ovules with a spatial range that depends on ovule incubation time.

**Conclusions:**

We propose a stochastic model that captures these dynamics. In the model, a pollen tube senses a difference in the fraction of receptors bound to an attractant and changes its direction of growth in response; the attractant is continuously released from ovules and spreads isotropically on the medium. The model suggests that the observed slowing greatly enhances the ability of pollen tubes to successfully target ovules. The relation of the results to guidance *in vivo *is discussed.

## Background

**Figure 1:**
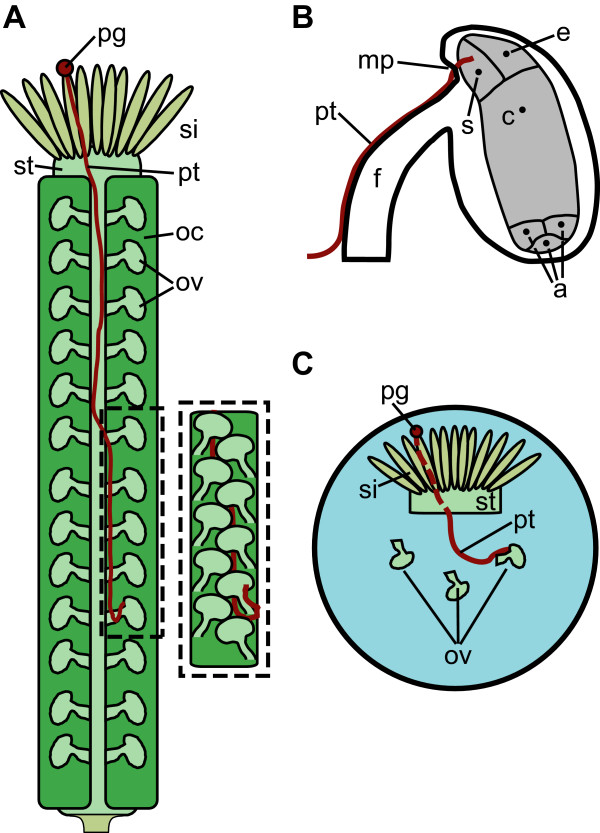
**Schematics of fertilization *in vivo *and *in vitro***. (A) Schematic depiction of the pollen tube path through the ovary. Dashed box shows growth between the rows of ovules after emergence in the ovary chamber. pg-pollen grain, pt-pollen tube, si-stigma, st-style, oc-ovary chamber, ov-ovules. (B) Schematic depiction of an ovule and a pollen tube approaching the micropyle. *In vivo*, pollen tubes extend along the funiculus, a cylindrical structure that connects the ovule to the placenta, and enter the ovule through the micropyle, an opening in the integuments that line the embryo sac. pt--pollen tube, f--funiculus, mp--micropyle, s--synergid cell, e--egg cell, c--central cell, a--antipodal cell. (C) Schematic depiction of semi-*in vitro *experiments with a cut style and dissected ovules. The pollen tube grows through the cut style (dashed portion), emerges and grows on the surface of the agar medium where it locates and fertilizes an ovule. For simplicity, only one pollen tube is depicted here. Abbreviations are the same as in A.

In flowering plants, unlike animals, the male and female germ units are multicellular, haploid structures that develop in different organs of the flower (Fig. 1A and 1B). In *Arabidopsis thaliana*, the male gametophyte, the pollen grain, comprises two sperm cells enclosed within a vegetative cell. The female gametophyte, the embryo sac, is a seven-cell structure that includes the egg cell and other haploid cells crucial for forming a viable seed; it is enclosed within maternal diploid tissue in an ovule (Fig. 1B). The sperm cells of flowering plants are non-motile and are transported through pollen tubes from the stigma to the embryo sacs (Fig. 1A and 1B). After a pollen grain contacts the stigma, it polarizes and develops a growing extension (the pollen tube) that traverses the pistil, eventually fertilizing an ovule by growing along its funiculus, entering through its micropyle (Fig. 1B), and releasing sperm cells into its embryo sac.

Many mechanisms have been proposed to explain how pollen tubes are guided to ovules, including mechanical tracts that direct growth, surface-expressed guidance cues, and diffusing signals [1-4]. *In vitro *experiments showed that *Nicotiana alata *pollen tubes use water as a directional cue in their initial growth through the stigma [5], and chemocyanin, a molecule released in the lily style, has been shown to induce chemotropism [6]. These observations suggest that following a gradient may play an important role in the earlier stages of pollen tube growth. Semi-*in vitro *investigation suggests that fertilized ovules may emit a short-lived repulsive signal to prevent multiple pollen tubes entering [7], and nitric oxide has also been shown to repel pollen tubes in *in vitro *[8] and semi-*in vitro *assays [9]. More recently, it has been shown that the synergid cells of *Torenia fournieri *secrete small peptides that induce chemotropism [10]. Although these observations provide evidence for diffusible attractants, the mechanisms of action of the participating molecules remain unknown, as do their identities in most species. Furthermore, a lack of detail in characterizing pollen tube responses has complicated discussions of the range at which the guidance operates and, in turn, the role of guidance *in vivo*.

A series of semi-*in vitro *experiments have provided substantial evidence that diffusible signals that are released by the ovule *in vitro *play a potentially important role in later stages of guidance. In these experiments, stigma are pollinated, cut, and placed on an agar medium [7,10-13]. Ovules are dissected from the ovary and arranged around the cut end of the stigma (Fig. 1C). The pollen germinates on the stigma, grows through the style, and emerges onto the surface of the medium. In *Gasteria Verrucosa*, *Torenia *and *Arabidopsis*, pollen tubes that emerged onto the medium showed an attractive response to the dissected ovules [7,11,12]. Semi-*in vitro *experiments in which cells in the embryo sac were systematically laser ablated revealed that the synergid cells are essential for this *in vitro *attraction in *Torenia *[14]. Both *Arabidopsis *and *Torenia *pollen tubes show less attraction to the ovules of closely-related species than to their own, and the amount of attraction decreases with evolutionary distance between the pollen tube species and the ovule species [7,13].

Here we present a quantitative analysis of newly obtained time-lapse images from such a semi-*in vitro *assay to investigate the mechanisms that mediate the attraction between pollen tubes and ovules in *Arabidopsis*. Our goal is to characterize systematically how pollen tubes sense and respond to the presence of ovules *in vitro*. To probe the dynamics of the interactions between pollen tubes and ovules, we varied the amount of time that dissected ovules had been incubated on medium relative to when the pollen tubes emerged from the cut style and grew toward the ovules. We found that pollen tubes show more attraction to ovules with longer incubation times, and that pollen tubes are attracted to ovules *in vitro *at distances of 100-150 *μ*m from the micropyle. This range of guidance is considerably longer than previously estimated [7]. Our analysis also indicates that pollen tubes decrease their rate of growth as they approach an ovule, and that this effect becomes stronger with longer ovule incubation times. Furthermore, pollen tubes often turned toward ovules, consistent with pollen tubes following a gradient of an attractant by sensing a change in the concentration of the attractant across their tips.

To explore the implications of these results, we developed a mathematical model of pollen tube response to a gradient of a diffusible attractant that is continuously released by the ovules. Because little is known about the receptors and internal signals that drive pollen tube response to such attractants, our model makes no assumptions about the molecular mechanism for sensing this gradient and instead focuses on whole-cell features, an approach which has been used to model algae phototaxis [15], whole-cell motility [16,17], trajectories of *Listeria *[18], and leukocyte chemotaxis [19-21]. The model successfully captures both the directed and random growth we observe experimentally and suggests that the observed slowing of growth *in vitro *greatly increases the ability of pollen tubes to target an ovule successfully. The implications that our observations and model have for guidance *in vivo *are discussed.

## Results

### Incubation time influences pollen tube response

Previous semi-*in vitro *work has shown that pollen tubes approach the micropyle of functional ovules more frequently than heat-treated ovules [11] or ovules with laser-ablated cells [14]. More recent approaches have quantified this apparent attraction by assessing how the rate of *in vitro *fertilization changes when pollen tubes are exposed to ovules dissected from closely-related species [7,13]. Here we present a quantitative analysis of how pollen tubes grow and respond to dissected ovules *in vitro*.

Dissected ovules from *Arabidopsis thaliana *plants were arranged around a cut style using a procedure adapted from [7] (see Methods). The cut styles were pollinated such that between 20-40 pollen tubes eventually emerged from the style onto the medium, where the tubes were then allowed to grow 30 minutes before imaging was started (Table 1). Confocal stacks were acquired every 20 minutes for 320 minutes. To assess pollen tube growth quantitatively, we tracked the positions of the pollen tube tips at each time point, and used these positions to construct trajectories of tube growth. These trajectories were combined with the locations of the micropyles of the ovules to give distance and angle data, and data from stigmas with the same incubation time were combined.

**Table 1:** Experimental details

		heat-treated	0 hours	2 hours	4 hours
Timing (hours)	Stigma	0	0	0	0
	Pollen	0	0	0	2
	Ovules	0	2	0	0
	Imaging	2.5	2.5	2.5	4.5
Count (number)	Stigmas	4	7	5	5
	Ovules				
	Penetrated/total	0/12	14/21	12/15	15/15
	Pollen tubes	149	223	175	132
Starting distance (*μ*m)		393.57 ± 17.85	415.68 ± 16.62	384.36 ± 20.26	430.16 ± 20.19

For various incubation times, these were the relative times that the stigmas were placed on the medium, the ovules were placed on the medium, the stigmas were pollinated, and imaging was started. Stigmas were always placed at 0 hours. The time for placing the ovules and for pollinating the stigmas was adjusted to give the ovules additional incubation time on the medium. Pollen tubes emerged 2-2.5 hours after pollination. For each incubation time, the total number of stigmas sampled, ovules penetrated and number pollen tubes analyzed is listed. The starting distance reported is the average distance between the center of the transmitting tract and the micropyles. These distances were not significantly different from each other (*p *> 0.1, one-way ANOVA).

**Figure 2:**
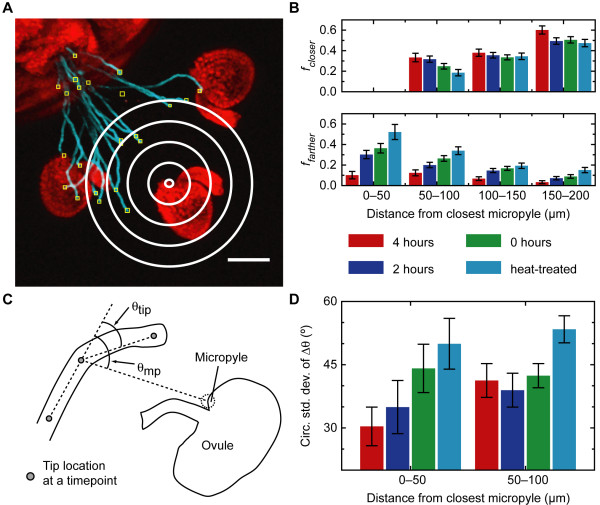
**Pollen tube attraction to ovules**. (A) Ovules and the cut stigma (upper left corner) are shown in red. Pollen tubes are shown, emerging from the style, in blue. The white concentric circles depict radial bins of 50, 100, 150, 200 *μ*m around one of the micropyles (central white circle). The tips of the pollen tubes are marked with yellow boxes. Scale bar (white) is 100 *μ*m. (B) Bar chart describing the time-averaged frequency that, at a given distance, the tip of a pollen tube grew closer to (top) or farther from (bottom) the nearest micropyle. The distances are split into radial bins with Δ*R *= 50 *μ*m (0-50 *μ*m, 50-100 *μ*m, etc.). (C) Depiction of *θ*_*mp *_and *θ*_*tip *_angles used in the analysis of pollen tubes turning. The *θ*_*mp *_angle indicates how much the pollen tube would have to turn to take the most direct path toward the micropyle. The *θ*_*tip *_angle describes the new direction chosen by the pollen tube in response to the gradient. (D) Circular standard deviations σ_0 _for distributions of Δ*θ *for points 0-50 *μ*m and 50-100 *μ*m from the closest micropyle for directions where the pollen tube is growing toward the micropyle (cos *θ*_*mp *_≥ 0). The key for the bars shown in B and D is the same.

To assay the amount of attraction that pollen tubes had toward an ovule, we calculated the fraction of pollen tube tips that were within a certain distance of a micropyle that grew either closer to (*f*_*closer*_) or farther from (*f*_*farther*_) that micropyle by the next time point (Δ*t *= 20 min). To this end, we measured the distance from the tube tip to the closest micropyle at each pair of adjacent time points *t *and *t *+ Δ*t *and constructed 50 *μ*m bins of these distances (Fig. 2A and 2B). The bin size of 50 *μ*m corresponds to the distance an average tube would grow in Δ*t *= 20 minutes, based on the previously reported rate of growth of 2.5 *μ*m/min [7]. We counted the number of tubes whose tips were in a bin at time *t *(*N*_*total*_) and how many of these tips had moved into either a closer bin (*N*_*closer*_) or a farther bin (*N*_*farther*_) at time *t*+Δ*t*. To assess the attraction of the pollen tubes over the course of the experiment, we combined these quantities for each bin over all time points into time-averaged frequencies that tips would move closer to or farther from an ovule in the time between confocal acquisitions: *f*_*closer *_= *N*_*closer*_/*N*_*total *_and *f*_*farther *_= *N*_*farther*_/*N*_*total*_.

Using this approach, we examined these frequencies for ovules that had incubated on the medium for 0, 2, and 4 hours. As a negative control, we used heat-treated ovules that had been incubated for 2 hours. This incubation time was chosen to be consistent with previous experiments in *Arabidopsis *[7]. Palanivelu and Preuss had placed heat-treated control ovules at the same time as pollinating the cut style, which corresponds to an incubation time of 2 hours in our assays (Table 1). In each experiment, the cut end of an ovary was placed a minimum of 250 *μ*m (typically 380-430 *μ*m) from the micropyle of an ovule; there was no signficant difference (*p *> 0.1, one-way ANOVA) between the average distances from the center of the cut transmitting tract to each micropyle in any of the experimental conditions (Table 1). We found that at all distances (0-200 *μ*m), the frequency with which tips moved farther from a micropyle of an ovule decreased with the incubation time of that ovule (Fig. 2B, bottom). The trends were very consistent: at all distances, the frequency of tips growing farther (*f*_*farther*_) from the micropyle of ovules that had been incubated for 4 hours was significantly different (*p *< 0.001) from both that of our heat-treated control and ovules that had been incubated for 0 hours (*p *< 0.01 for distances of 0-150 *μ*m and *p *< 0.05 for 150-200 *μ*m). Compared to the strong effect of incubation time on *f*_*farther*_, the effects of incubation time on *f*_*closer *_were less visible (Fig. 2B). This difference stems from the facts that pollen tubes persist growing in the same direction for long distances, and the direction of the cut style initially orients the tubes to grow toward the ovules in the semi-*in vitro *assay.

The previous statistics include pollen tube growth that occurs both before and after the pollen tube penetrates the ovule. The points after penetration were included to allow an unbiased comparison with the heat-treated control but may affect the trends in *f*_*closer *_and *f*_*farther*_. To prevent polyspermy, the interactions between pollen tubes and ovules change once an ovule is fertilized, which occurs shortly after pollen tube penetration [7,22-25]. We constructed frequencies  and  for functional ovules that only include points in each pollen tube trajectory that and *f*_*farther *_correspond to times before the nearest ovule was penetrated. The trends in these frequencies were consistent with those reported above for *f*_*closer *_and *f*_*farther*_, although the differences between the three incubation times in  were not significant (data not shown).

Effectively, *f*_*farther *_quantifies the degree to which ovules cause pollen tubes to deviate from random growth once they come within a certain distance of a micropyle, but this statistic does not address whether growth while approaching this region is directed. To further analyze pollen tube approach, we defined two angles: *θ*_*mp *_and *θ*_*tip*_. The angle *θ*_*tip *_is the angle that a pollen tube turns as it grows, and the angle *θ*_*mp *_is the angle that a pollen tube would have to turn to grow directly toward the micropyle (Fig. 2C). The difference between these two angles, Δ*θ *= *θ*_*mp *_- *θ*_*tip*_, measures how much pollen tube growth deviates from the most direct path toward the micropyle (Δ*θ *= 0°). Owing to the periodic nature of angles, the distribution of Δ*θ *cannot be characterized by the usual descriptive statistics of mean and standard deviation [26,27]. Instead, we treat each angle as a unit vector on a circle, and use the average direction and average length of these vectors to compute a circular mean and a circular standard deviation (see Methods). To characterize how pollen tubes approached ovules, we limited the angles in this characterization to cos *θ*_*mp *_≥ 0.

We use these statistics to summarize how different incubation times affected the deviation in guidance represented by the Δ*θ *angle for pollen tubes with tips 0-50 *μ*m and 50-100 *μ*m from a micropyle (Fig. 2D). As previously described, care was taken to ensure that conclusions were based on functional ovules (see Methods). At distances of 0-50 *μ*m, the mean angle ⟨Δ*θ*⟩ was not significantly different from 0° under any of the conditions. However, the circular standard deviations (*σ*_0 _decreased with the incubation time, and the heat-treated control had the widest distribution (Fig. 2D). At distances of 50-100 *μ*m, the mean angle ⟨Δ*θ*⟩ was only significantly different from 0° (*p *< 0.05) for pollen tubes approaching ovules that had been incubated for 0 hours, where ⟨Δ*θ*⟩ = 10.9 ± 5.2°. At these distances, there was no significant difference in the circular standard deviations *σ*_0 _of the functional ovules, but all three were significantly different (*p *< 0.01) from the behavior of pollen tubes approaching heat-treated ovules (Fig. 2D).

In each experiment, the pollen tubes grew similar distances before reaching the ovules, which indicates that the difference in response results from the ovule incubation time. These data support a model where ovules release a diffusible signal (attractant) throughout the experiment, independently of the presence of pollen tubes. The data also suggest a putative range over which the response operates: both the frequency *f*_*closer *_and the distribution of the angle Δ*θ *shows that pollen tubes that grow within 50-100 *μ*m of the micropyle of an ovule show an increased reorientation to that ovule. Furthermore, within 0-50 *μ*m, pollen tubes appear to be more directly guided to ovules with longer incubation times. Although the operative range of attraction *in vitro *may vary with different experimental conditions, this range of 100 *μ*m is larger than the value of 33 ± 20 (s.d.) *μ*m, which was based on observing when tubes made sharp turns toward the micropyle under similar agarose preparations [7].

### The pollen tube response is consistent with following a gradient

**Figure 3:**
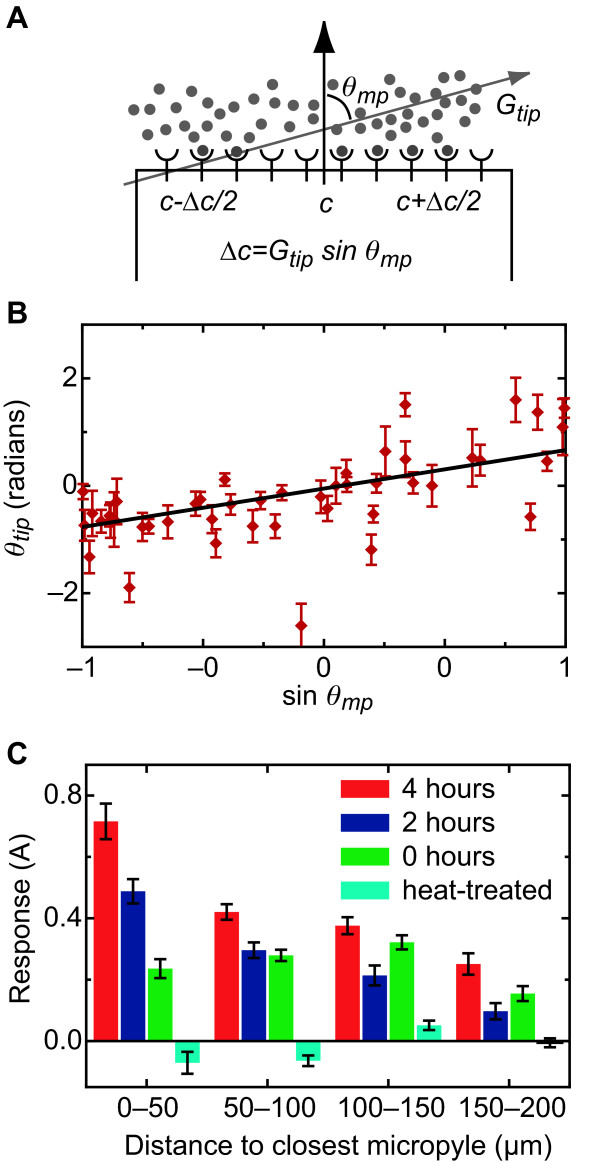
**Pollen tube behavior is consistent with turning in response to a gradient of an attractant across the tip surface**. (A) Schematic of gradient-following model. The pollen tube tip is treated as flat. A gradient in the attractant (*G*_*tip*_) concentration gives a difference in concentration Δ*c *between the two sides of the tip. (B) Fit of *θ*_*tip *_= *A*sin *θ*_*mp *_+ *ε *for points 0-50 *μ*m from the micropyle and 4-hour incubation time. In all fits, *ε *was not significantly different from zero. The slope *A *can be considered the average response of the pollen tubes to the ovule. (C) Fits were obtained at varying distances from the closest micropyle: 0-50 *μ*m, 50-100 *μ*m, 100-150 *μ*m, and 150-200 *μ*m. The turning response (the slope *A*) measures the average tendency for pollen tubes to turn toward the micropyle based on the hypothesis that the turns sense a change in the concentration of an attractant across the tip. Turning responses are given for data collected with 0-, 2-, and 4-hour ovule incubation times and also for heat-treated (boiled) ovules. Error bars are the standard errors determined by the linear regression.

Previous studies have focused their analysis on only the sharp, obvious turns that pollen tubes make near the micropyle, both *in vivo *[22] and *in vitro *[7]. Here we define a quantitative metric (the turning response) that assesses the mean turning behavior of the pollen tubes for both large turns and more subtle turns. To define a turning response, we measure the correlation between the turns that the tube makes and the direction toward the micropyle (*θ*_*tip *_and *θ*_*mp *_in Fig. 2C, respectively). Because diffusion of a released attractant should be approximately isotropic on the surface of the medium, the direction of the gradient is expected to be along the angle *θ*_*mp*_. In *Dictyostelium discoideum *and other eukaryotic cells undergoing chemotaxis, small GTPase proteins are thought to be intermediaries between the receptors that bind to chemokines and events in the cytoskeleton that effect chemotaxis [28]. Based on studies of Rop GTPase, a Rho-like GTPase that is localized in pollen tube tips [29] and that marks the site of tube growth [30], we assumed that the receptors involved in pollen tube guidance are primarily localized near the tip of the tube. If *G*_*tip *_is the magnitude of the gradient at a tip, Δ*L *is the width of the tip, and Δ*c *is the difference in concentration across it, Δ*c*/Δ*L *= *G*_*tip *_sin *θ*_*mp *_(Fig. 3A), where *G*_*tip *_is in units of the change of concentration per unit distance. If a pollen tube is following a gradient of attractant, then its turns should be correlated with Δ*c*/Δ*L*, and thus sin *θ*_*mp*_.

To test this hypothesis, we looked at the relation between *θ*_*tip *_and sin*θ*_*mp *_by fitting the line *θ*_*tip *_= *A *sin *θ*_*mp *_+ *ε *(Fig. 3B) for the turns pollen tubes made at different distances from the micropyles of ovules that had been incubated for different times (Fig. 3C). In each case, there was a significant relation, as measured by the Pearson *r *values and the slopes of the regression lines (Table 2), at 50-100 and 100-150 *μ*m from the micropyle of ovules incubated for 0, 2, and 4 hours. At distances of 150-200 *μ*m, there were still significant correlations (*p *< 0.05) for ovules incubated for 2 and 4 hours. As expected, datasets for the heat-treated ovules did not show significant correlations. In all cases, the intercept *ε *was not significantly different from zero. These results are consistent with a mechanism where a pollen tube follows a gradient of the attractant by turning in response to sensing a difference in the attractant concentration across its tip.

**Table 2:** Pollen tube turning responses.

	Distance (*μ*m)	Response	ΔResponse	Pearson r	p-value (%)	
0 hours	0-50	0.236	0.031	0.28	2.49	•
	50-100	0.279	0.018	0.37	3.5 × 10^-3^	••••
	100-150	0.322	0.0231	0.6	1.0 × 10^-5^	••••
	150-200	0.155	0.024	0.22	6.74	
2 hours	0-50	0.488	0.04	0.48	0.17	••
	50-100	0.296	0.025	0.55	1.1 × 10^-3^	••••
	100-150	0.214	0.032	0.35	1.06	•
	150-200	0.097	0.026	0.29	2.52	•
4 hours	0-50	0.716	0.058	0.65	0.01	•••
	50-100	0.42	0.025	0.56	3.3 × 10^-4^	••••
	100-150	0.376	0.028	0.55	2.1 × 10^-3^	••••
	150-200	0.251	0.035	0.46	0.21	••
heat-treated	0-50	-0.071	0.036	-0.091	54.97	
	50-100	-0.064	0.017	-0.08	32.52	
	100-150	0.051	0.015	0.112	9.16	
	150-200	-5.7 × 10^-3^	0.015	0.027	68.39	

Responses reported are the unitless slope *A *of the regression line between *θ*_*tip *_and sin *θ*_*mp*_. The column ΔResponse is the standard error of this slope. The significance levels reported are for the Pearson *r *values: *p *< 5% (•), *p *< 1% (••), *p *< 0.1% (•••), *p *< 0.01% (••••).

These correlations provide an estimate of the range of response that is consistent with our previous *f*_*closer*_/*f*_*farther *_and Δ*θ *analyses. The correlations at 0-50 *μ*m, 50-100 *μ*m, and 100-150 *μ*m are significant: each Pearson *r *has a probability of occurring randomly of *p *< 0.05, and often *p *< 0.001, and the slopes of the regression lines (*A*) are different from zero with similar statistical significance. The correlations at distances of 150-200 *μ*m are smaller and less significant, and occur at the largest distances in our analysis. Our analysis suggests that pollen tubes respond to ovules at distances at least as far as 150 *μ*m, although the response at larger distances was often smaller than the random turns pollen tubes made. In addition to allowing us to infer the range of the response, the slope *A *of each regression model (Fig. 3C), is a measure of the pollen tube response at that distance and incubation time, and also provides an estimate of the size of the gradient of the attractant (i.e., *A *~ *G*_*tip*_). The data evidence two trends for this response: it increases with longer incubation times and decreases at farther distances from the micropyle (Fig. 3C).

Although pollen tubes are known to turn in response to changes in their internal tip-focused cytoplasmic calcium gradient [31], and gradients of small molecules (ions and reactive species) affect pollen tube polarity and influence the direction of growth [8,31-34], the mechanisms that couple external guidance cues to these intracellular ion gradients remain unknown. Both spatial and temporal sensing mechanisms have been suggested in the literature on pollen tube guidance [1]. Our analysis supports a spatial mechanism in which the pollen tubes effectively measure the concentration of the attractant across their tips and turn accordingly. In the temporal sensing that is characteristic of *E. coli *chemotaxis, a bacterium displays a series of runs that are separated by isotropic tumbles [35-37]. This mechanism is inconsistent with our findings, and would be hard to reconcile with the smooth growth that pollen tubes undergo. However, our results do not rule out more complex guidance mechanisms that could modulate how pollen tubes follow a gradient based on some memory of previous concentrations or gradients [38].

### The turns pollen tubes make are well-described by a model where ovules continuously release an attractant and pollen tubes respond to this attractant by following its gradient

Our experimental results show that pollen tubes change their direction of growth in a manner consistent with responding to a change in concentration across their tip, and that this response increases both with longer incubation times and as pollen tubes grow closer to the micropyle. To test and refine the mechanisms suggested by these data, we developed a mathematical model that encompasses both the release of an attractant by the ovules and the subsequent response that pollen tubes have to the attractant. Existing models of pollen tube behavior have focused on the physical processes that underlie tube growth, where cell shape, turgor pressure, internal ion gradients, and vesicle trafficking are essential considerations. Most models describe general tip growth in plants and fungi [39-42], although some recent work incorporates specific details of the pollen tube [43,44]. Because little is known about the molecular mechanisms that mediate interactions between pollen tubes and ovules, we kept the model minimal. Despite the lack of molecular detail, our model captures both the directed and random growth in pollen tube guidance and aids interpretation of the experimental results.

**Figure 4:**
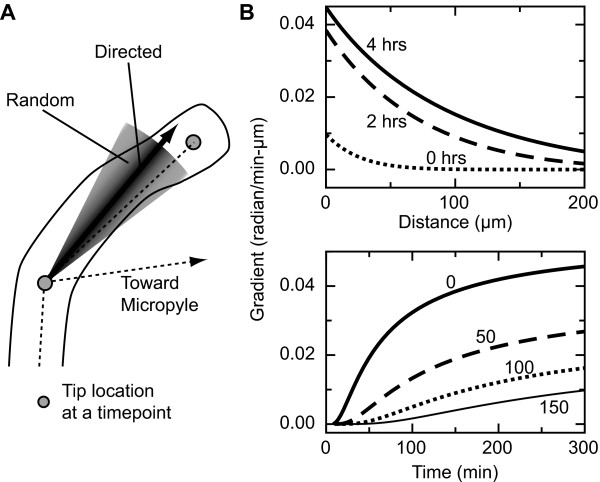
**Model of pollen tube growth**. (A) Conceptual depiction of the directed and random components of turning. The directed component (black arrow) is calculated based on the gradient of the attractant. The random component is a random angle added to this. The gray shaded regions depict one standard deviation of the Gaussian distribution for the random angle. (B) Dynamics of a model of the gradient. The model gives a theoretical concentration of the attractant (Eq. 3 in Methods), and the gradient is derived from this concentration. Here the magnitude of the gradient from a single ovule, oriented toward the ovule micropyle is shown. Top: Depiction of the model for the attractant gradient as a function of distance from the micropyle. The different curves (top to bottom) are for the gradient after the source has released the attractant for 4.5 hours, 2.5 hours, and 0.5 hours. Bottom: Depiction of the model for the attractant gradient as a function of time on the medium. The different curves (top to bottom) are for distances of 0 μm, 50 μm , 100 μm , and 150 μm from the micropyle.

We modeled how pollen tubes change their direction of growth by splitting each turn into a directed and a random component (Fig. 4A), which we assumed were independent. The directed component specifies the mean angle that a theoretical pollen tube would turn in response to a gradient of the attractant, and the random component adds a random angle chosen from a Gaussian distribution to this mean direction. To determine the directed component, we assume that each bound receptor induces a signal that gives the pollen tube some propensity to turn in the direction of the receptor. For a pollen tube to perceive a difference in the concentration across its tip, there must be at least two patches of receptors that are spatially separated on the pollen tube. Similar simple considerations have led to several successful models of leukocyte chemotaxis (for example, [20,21]).

An exact model of spatial sensing would depend on both the distribution of receptors in these patches (or across the whole tip), the kinetics of the receptor-ligand interaction, and the nature of the intracellular response that ultimately results in the pollen tube turning. The distance and time scales in our experiment are large enough that we can assume receptors operate close to steady-state. We simplify the remaining considerations by assuming that the change in concentration across the tip (Δ*c*) is much less than the average concentration at the tip (*c*), in which case both the concentration along the tip and the difference in bound receptors are approximately linear. The directed component can then be approximated as proportional to the difference in the receptors bound between the left and right ends of the tip. The trends in Fig. 3B do not indicate any saturation; furthermore, initial fits of our data to this case further suggested that the directed component was well modeled by receptors far from saturation, where the ligand binding is stoichiometric. In this regime, the directed component of turning is then proportional to the difference in concentration across the tip, making our model of turning(1)

where Δ*c *is the difference in concentration across the tip, and *κ *is the proportionality constant.

To relate this model to the data in our experiments, we introduced a model for a relative concentration profile of the attractant (Fig. 4B, Eq. 3 in Methods). This profile evolves by two processes: release of the attractant at the micropyle and diffusion of the attractant on the artificial medium. Because the details of how ovules release the attractant are not known, we model this release in a way that is consistent with our observations of the pollen tube response: the local concentration of the attractant and, more importantly, its gradient should increase both with longer incubation times and as the pollen tubes grow closer to the micropyle. The increase in the gradient at longer incubation times implies ongoing release at the source [45-47]. To simplify the description of diffusion on the medium, we considered only two-dimensional diffusion through the thin fluid film that coats the surface of the medium and not through the agar matrix itself.

Modeling the difference in concentration across the tip of the pollen tube requires relating how the concentration at the tip changes as the position of the tip changes. As discussed in Section 2.2, we expect Δ*c*/Δ*L *= *G*_*tip *_sin *θ*_*mp *_(Fig. 3A). Consistent with our experimental observations, *G*_*tip *_decreases with distance (the turning response increases closer to the micropyle) and increases with time (the turning response increases with longer incubation times).

**Figure 5:**
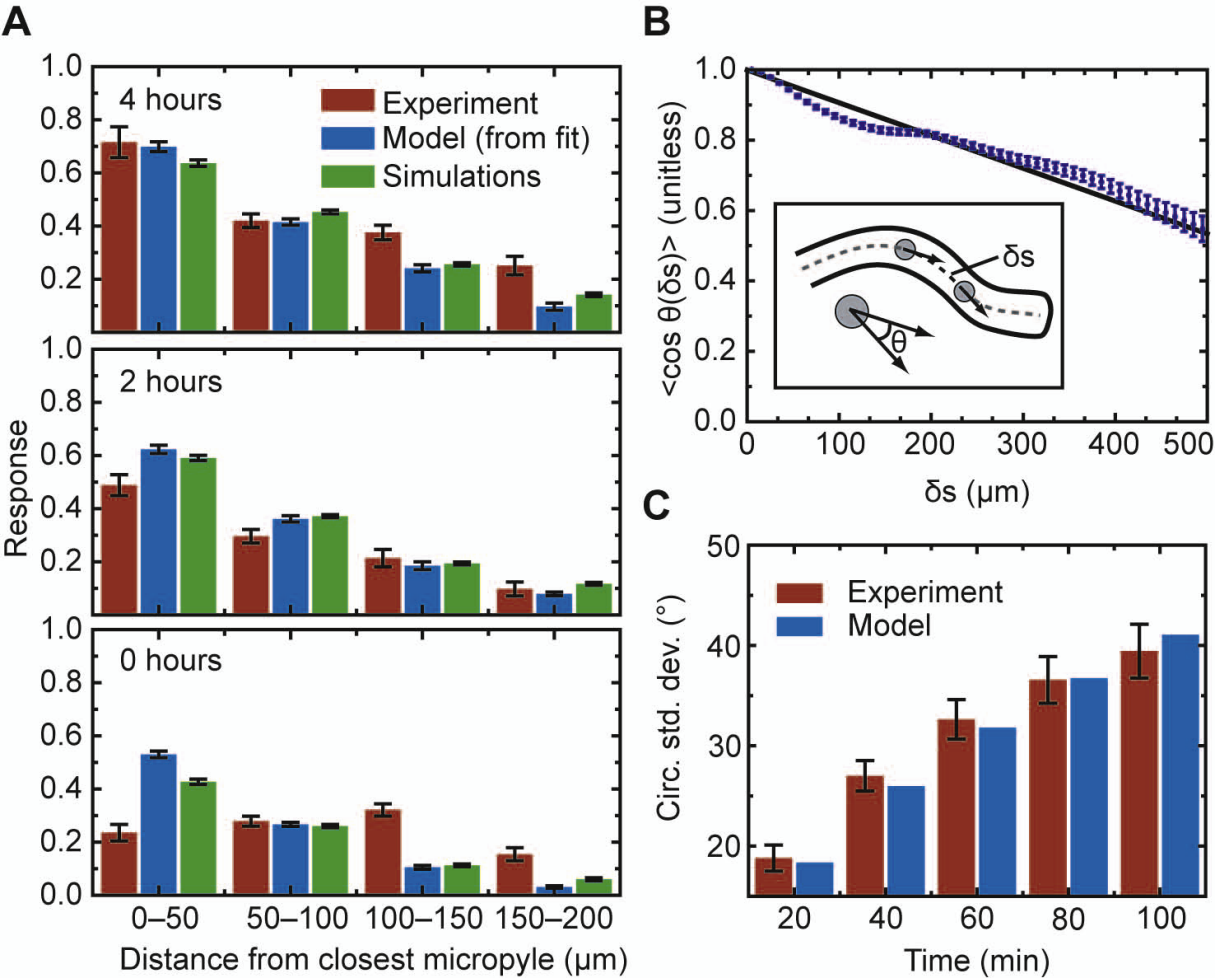
**Validating the model**. (A) Comparison of experimental results with the model. The responses are defined as in Fig. 3, where the response is the slope of the regression line between the turning angle *θ*_*tip *_and sin *θ*_*mp*_, which projects the gradient onto the tip of the pollen tube (see Fig. 3A). The different bars compare pollen tube responses observed in experiment, predicted from the model fit, and produced by simulations of the model. (B) Mean ⟨cos*θ*_*tip*_⟩ plotted against *δs*. We use a linear model to describe this relationship. Inset: Schematic depicting analysis of persistence length, used to set the model parameter *σ*. The distance between the two points along the tube path is *δs*, and the angle between their directions of growth is *θ*_*tip*_. The cosines of these angles are averaged for all points along the path separated by *δs*, and over all tube paths, giving the mean ⟨cos*θ*_*tip*_⟩ as a function of *δs*. (C) Comparison between the circular standard deviations (*σ*_0_) predicted from the linear fit in panel B, *σ*^2 ^= 2*vδt*/*L*, and the actual values for pollen tubes growing in the absence of ovules. The comparison is plotted for several time intervals. The growth rate, *v*, was set to 2.76 *μ*m/min.

When we combine the model for the direction of pollen tube growth and the attractant gradient, there are four parameters that describe the mean direction that the tubes turn in response to an attractant: the turning constant (*κ*), the rate of attractant production (*k*_*p*_), the attractant diffusion constant (*D*), and an effective distance that accounts for diffusion of the attractant within the micropyle and on the ovule surface before its deposition onto the medium (*r*_0_). However, the parameters *κ*, *k*_*p*_, and *D *are covariant (see Methods), and we used an effective turning constant *κ*' = *κ*(*k*_*p*_/*D*) in addition to *D*, and *r*_0 _as fitting parameters (Table 3). The resulting (deterministic) model shows reasonable agreement with experimental responses both close to and far from the micropyle (Fig. 5A), although it performs noticeably worse for 0-hour incubation times and at longer distances in 4-hour incubation times.

**Table 3:** Parameters for the turning model

Parameter	Description (units)	Fit value	90% CI
*κ*'	Proportional response (rad/conc min)	40.11	34.50-63.91
*D*	Diffusion constant (*μ*m^2^/min)	66.72	63.63-96.69
*r*_0_	Radial offset (*μ*m)	117.56	116.01-174.61

The fit yields a diffusion constant of 66.72 *μ*m^2^/min, or 0.11 × 10^-7 ^cm^2^/sec (Table 3). The molecular weights of the attractants identified in *Torenia *[10] are approximately the same as that of ubiquitin, (8-9 kD), which has a diffusion constant of 14.9 × 10^-7 ^cm^2^/sec in aqueous solution [48]. Comparing the values is complicated by the high sucrose content of the thin film on top of the medium (18% w/v) and the possibility of non-specific interactions between the attractant and the supporting agar. Both of these factors would decrease the rate of diffusion of the attractant. Given these considerations, the estimated diffusion constant is consistent with the attractant being a small to medium sized peptide. Previous *in vitro *studies have bound the molecular weight to 10-85 kDa by alternative means [7].

### Deviations from the mean direction of turning are consistent with how pollen tubes turn in the absence of ovules

Up to this point, our analysis has been used to understand the mean response of pollen tubes to the attractant, which is presumed to be released by ovules. We now turn to studying the substantial variation in response that pollen tubes exhibit [49,50]. Similar variation has been observed in many eukaryotic systems undergoing chemotaxis [19,21,51,52], and it is thought to be advantageous for cells that are seeking nutrients or other targets but have not yet detected them [52]. In our model, the variation is set by a persistence length, which specifies how much a tube would elongate before losing a significant component of its original direction. We assayed this length by analyzing trajectories of 58 pollen tubes in semi-*in vitro *assays where no ovules were added to the medium. The change in direction of a pollen tube was measured by the angle between the direction of growth at some distance along the tube *s *and a new direction of growth after the tube had grown a distance *δs *(Fig. 5B Inset). The correlation between these two points is mathematically equivalent to ⟨cos *θ*_*tip *_(*s, s *+ *δs*)⟩. Plotting this quantity as a function of *δs *shows that it is approximately linear, and regression yields an estimate for the persistence length of *L *= 1042.70 *μ*m. The long persistence length indicates that the probability of making a turn *θ*_*tip *_peaks sharply around *θ*_*tip *_= 0, such that ⟨cos *θ*_*tip*_⟩ ≈ 1 - ⟨*θ*_*tip*_^2^⟩ and that the probability distribution can be described as a sharply-peaked Gaussian with variance ⟨*θ*_*tip*_^2^⟩ = 2 (*δs/L*) (see Methods). The standard deviations predicted by this form compared well with the circular standard deviations of the actual angle distributions for Δ*t *= 20 to Δ*t *= 100 min (Fig. 5C).

Above, we assumed that the random component of growth is independent of the concentration of attractant. To test this idea, we ran simulations of our model that included both directed and random growth, with ovule locations and initial pollen tube locations and directions of growth taken directly from the corresponding experiments. We then treated these simulations as artificial time-lapse data and analyzed them in the same way that we analyzed our experimental data (see Methods). We found that the mean responses (directed component) in the simulations, as measured by the slope of the regression line between *θ*_*tip *_and sin *θ*_*mp*_, compared well to the data at different distances and for different incubation times (Fig. 5A). We also assessed whether the random growth seen in our simulations was comparable to that in the experiments by analyzing the residuals, differences between the *θ*_*tip *_predicted by the regression and the actual *θ*_*tip*_. We compared the standard deviations of the populations of these residuals for both the simulations and the experiments (Table 4). The standard deviations showed good agreement at distances far from the micropyle (150-200 *μ*m), where the effects of an ovule should be small, and also matched at closer distances (100-150 *μ*m) where there was a measurable response to the ovules. At even closer distances (50-100 *μ*m), the standard deviations compared well for 2-hour incubation times and reasonably well for 4-hour incubation times, but the experimental data had larger standard deviations at 0-hour incubation times than did our simulations. At the closest distances (0-50 *μ*m), the standard deviations of the experiments were much larger than those of the simulations. This difference resulted largely from outliers in the distribution, as indicated by the fact that the standard deviations of a data set with points outside twice the inter-quartile range removed showed much better agreement. However, the difference could also indicate that the gradient changes more rapidly at these close distances than can be captured using our linear model for the turning response (Fig. 3).

**Table 4:** Comparison of variations in responses in experiments and simulations.

	Distance (*μ*m)	Experiment (radians)	Simulation (radians)
0 hours	0-50	0.747 ± 0.083	0.283 ± 0.006
	50-100	0.550 ± 0.051	0.278 ± 0.003
	100-150	0.324 ± 0.047	0.271 ± 0.003
	150-200	0.335 ± 0.085	0.269 ± 0.003
2 hours	0-50	0.657 ± 0.078	0.306 ± 0.006
	50-100	0.332 ± 0.029	0.291 ± 0.003
	100-150	0.314 ± 0.036	0.281 ± 0.003
	150-200	0.235 ± 0.023	0.268 ± 0.003
4 hours	0-50	0.602 ± 0.100	0.311 ± 0.007
	50-100	0.420 ± 0.038	0.288 ± 0.004
	100-150	0.383 ± 0.054	0.283 ± 0.003
	150-200	0.264 ± 0.025	0.272 ± 0.003

Comparison of the circular standard deviations of turns in simulation and experiment. This summarizes the deviations from the mean turning response, which we treat as the random component of growth. This random component was calculated from the residual deviations between the mean response and the individual responses.

### Incubation time influences the rate of growth near the micropyle

**Figure 6:**
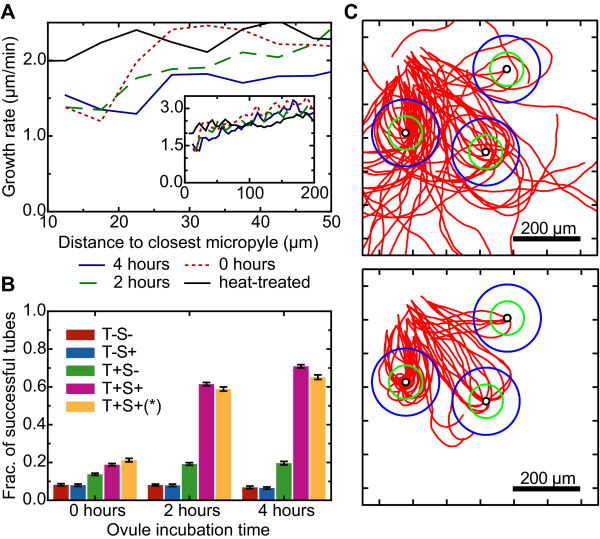
**Analysis of the growth rate near the micropyle**. (A) The average rate of growth depends on the distance to the micropyle. When pollen tubes grow within 50 *μ*m of functional ovules, they consistently slow their rate of growth, an effect not observed with heat-treated ovules. When pollen tubes approach ovules with a longer incubation time, this slowing occurs at longer distances. (Inset) The growth rates of functional and heat-treated ovules are consistent until pollen tubes grow within 50 *μ*m of a micropyle. (B) The fraction of tubes able to target micropyles in simulations where either the turning response (T) or the slowing response (S) were removed to determine the role each would play in guidance. The set T+S+ (*) indicates simulations were the random growth was kept constant, and did not show a significant difference in targeting compared to T+S+, where the size of the deviations in the random growth varied with the growth rate (). We considered a pollen tube to have targeted (fertilized) an ovule successfully when its tip reached a 10 *μ*m distance from a micropyle. (C) Characteristic paths of tubes at 4-hour incubation times in the T+S- simulations (top) and T+S+ simulations (bottom). The positions of the ovules, and the positions and initial directions of the pollen tubes were taken from the experiment shown in Fig. 2A.

When we measured the persistence length of pollen tubes, we observed that the tubes began growing with an average rate of 2.76 ± 0.05 *μ*m/min, consistent with previously reported values [7]. This rate slowed to 1.0-1.5 *μ*m/min after the tubes had grown for 4 hours, both with and without ovules. While [7] observed that pollen tubes decreased their rate of growth as they approached the micropyle, they did not distinguish this effect from the gradual slowing that generally occurs in the semi-*in vitro *assay. Consequently, we examined how the average rate of growth changed at different distances to the micropyle for both functional and heat-treated ovules. The growth rates were calculated by dividing the distance between adjacent points in the time-lapse data by the time between those measurements (20 min). We considered the distance between the first of these points and the closest micropyle as the distance to the micropyle. Average rates of growth were calculated at 5 *μ*m intervals for distances of 10-200 *μ*m, and points within 5 *μ*m of the interval center were included in the average to reduce noise and help visualize the resulting trends. We found that when pollen tubes approached functional ovules, their rate of growth substantially decreased. This decrease was not present when pollen tubes approached heat-treated ovules, and the incubation time of the ovules influenced this decrease by increasing the distance at which this slowing began (Fig. 6A). Specifically, within 50 *μ*m of the micropyle of heat-treated ovules, pollen tubes grew at a rate of 2.29 ± 0.08 *μ*m/min ; this rate of growth decreased with the incubation time of functional ovules, to 1.67 ± 0.11 *μ*m/min around ovules incubated for 4 hours (*p *< 0.001). Pollen tubes that approach ovules with 0-hours of incubation did not show a decrease in growth until very close to the micropyle, while the decrease was apparent at a larger distance for ovules with 2- and 4-hour incubation times. The slowing partially explains the difference in observed *f*_*farther *_frequencies at 0-50 *μ*m.

### In simulations, reducing the rate of growth increased the ability of pollen tubes to target ovules

To explore how this reduced growth rate would influence the guidance process, we added terms to our simulation to decrease the rate of growth with an increase in the gradient of the attractant (see Methods). These terms do not assume any particular molecular model for why the pollen tube slows, but their inclusion allowed us to include or exclude either a turning response (Eq. 1) or a slowing response (Eq. 4, in Methods). To assess the role that turning and slowing play in guidance, we calculated the fraction of tubes that were successfully able to target ovules in simulations for tubes that included or excluded these terms (Fig. 6B). About 6-8% of pollen tubes with no turning or slowing (T-S-) were still able to target the micropyle randomly, and when slowing was enabled (T-S+), this frequency did not change significantly. When turning was enabled with no slowing (T+S-), the frequency of tubes that would successfully target more than doubled (from 6% to 20% with a 4-hour incubation time), and this frequency increased to over 60% when both were enabled (T+S+). The difference between T+S- and T+S+ was visually striking, in that tubes that reduced their rate of growth showed substantially more guidance to the micropyle than tubes that had a constant rate of growth (Fig. 6C). Because the size of the random turns in our model varies with the growth rate, we also simulated pollen tubes whose rate of growth decreased with larger gradients of the attractant, but whose random turns stayed the same size ( was initially calculated for a growth rate of 2.76 *μ*m/min, but was kept constant). In these simulations (T+S+ (*)), the fraction of tubes that were successfully able to target ovules was close (differing by less than 5%) to those where the random deviations varied with the rate of growth (Fig. 6C), indicating that the greater guidance we observed in simulations where the growth rate decreases is a result of the growth rate and not of smaller random deviations.

These results assumed the same turning response, which indicates that slower pollen tube growth near the micropyle can increase the ability of the tube to correctly target the micropyle without requiring the mechanisms that drive turning (the receptors at the tip) to increase their sensitivity. This effect can be understood geometrically by calculating how much a pollen tube must turn to prevent the angle *θ*_*mp *_from increasing as it grows. Consider a pollen tube that grows at a rate *v *toward the micropyle, but not directly toward it (*θ*_*mp *_> 0°). If the pollen tube does not turn, over short times (*δt*), the angle *θ*_*mp *_increases by *δθ*_*mp *_= (*v*/*r*)sin(*θ*_*mp*_)*δt*, where *v *is the growth rate and *r *is the distance from the micropyle. Thus the tube must turn toward the micropyle by more than this amount to decrease *θ*_*mp*_. For a turning response of *A*, *δθ*_*tip *_= *A *sin(*θ*_*mp*_)*δt*, such that the total change in the angle *θ*_*mp *_is(2)

By this reasoning, the angle *θ*_*mp *_will only decrease (*δθ*_*mp *_< 0) when the response to the gradient *A *is larger than *v*/*r*. Eq. 2 shows that the pollen tube can increase its targeting by either increasing its turning response (*A*) or keeping a constant turning response and reducing its rate of growth (*v*).

## Discussion and conclusions

Previous semi-*in vitro *studies [7,10-14] have shown that a diffusible attractant plays a role in pollen tube guidance. Our quantitative analysis of *in vitro *pollen tube growth reveals a much longer range of pollen tube response in *Arabidopsis *than previously reported [7]. Our results suggest that this observed attraction *in vitro *results from a pollen tube sensing and responding to a difference in the concentration of attractant across its tip. Both the strength of the pollen tubes' response and their rate of growth were affected by the incubation time of the ovules and the distance of the pollen tube tips from the micropyle. Based on these data, we constructed a mathematical model of pollen tube growth. In the model, we assumed that ovules continually release an attractant and that it then diffuses on the medium. In the model, pollen tubes make turns that, on average, follow the gradient of this attractant, but they deviate from this path consistent with the random growth observed when pollen tubes are grown with no ovules present. This model successfully captures both the directed and random behavior of the pollen tubes growing *in vitro *and reveals that slowing growth near the micropyle can greatly aid fertilization.

Although the recent identification of pollen tube attractants in *Torenia *[10] is a significant step toward understanding the molecular mechanism of guidance, much still remains unknown. In particular, little is known of the molecular mechanisms within the pollen tube that enable sensing and responding to this attractant. Recent work on axon guidance identified an optimal means of integrating signals from multiple receptors [53]; in this model and the experiments to validate it, the response depends in a complex way on both the concentration of the attractant and the steepness of the its gradient. The authors suggest a number of possible molecular mechanisms that could give rise to this behavior, and these may also be relevant to pollen tubes. A more complex relation between the response and the concentration and steepness of the gradient could also explain why there is no indication of receptor saturation in our analysis, but more precise control of the attractant gradient would be required to validate this hypothesis.

Our model of the turning response did not assume a particular molecular mechanism for the sensing process. Some molecular features of pollen tube growth are likely to be important to include in future models as appropriate data become available. Pollen tubes have been observed to reorient in response to changes in the tip-focused cytoplasmic calcium gradient [31]. In *Dictyostelium discoideum *and other eukaryotic cells that undergo chemotaxis, GTPases are thought to link chemokine reception with the behavior of the cytoskeleton [28]. The protein Rop, a Rho-like GTPase, has been shown to localize to the tip of the growing tube [29], and its dynamics appear to lead the growth of pollen tubes [30]. Understanding how the attractant influences Rop and the tip-focused calcium gradient may provide further insight into the directed and random growth that pollen tubes undergo.

Some features of pollen tube growth are unique among studied chemotropic responses. The eukaryotic cells commonly used in studies of chemotaxis, *Dictyostelium discoideum *cells and leukocytes, are large (~10 *μ*m diameters). The pollen tubes of *Arabidopsis *have narrower widths (~5 *μ*m in *Arabidopsis*). Although they may effectively increase this width with receptors not confined to the tip of the tube, the growth machinery appears to be largely confined to the tip [31,32,54]. The small size (~1 *μ*m in length) of *E. coli *is thought to dictate its use of a temporal sensing mechanism for chemotaxis [35,55,56]. *Dictyostelium *cells and leukocytes sense a spatial gradient and polarize in response to this gradient before becoming motile [28], which allows the cells to respond to gradients almost isotropically. In contrast, pollen tubes secrete a cell wall as they grow and can only change direction by apical extension at the tip. This mechanism of growth enforces an internal polarity that renders pollen tubes unable to respond isotropically to a gradient, but this natural polarity may decrease the size required to efficiently sense gradients, perhaps by time-averaging of the number of bound receptors.

Our results support a long-ranged chemotropic model for pollen tube guidance where pollen tubes respond to stable gradients maintained by ovules continuously releasing an attractant. Similar mechanisms have been proposed in genetic studies of the guidance process, but it is unknown how the attraction observed *in vitro *will manifest *in vivo*. Recent genetic studies have uncovered mutants that suggest a two-stage model for guidance in the ovary [22,57-60]. In this model, pollen tubes show a short-range of attraction near the micropyle (micropylar guidance) that is distinct from the longer-range guidance that attracts them to grow along the funiculus (funicular guidance) [22].

The range of attraction *in vitro *(100-150 *μ*m) is roughly the same as the range of attraction required of funicular guidance *in vivo*. However, differences between the *in vitro *and *in vivo *environments make these distances hard to compare. Our results strongly suggest that pollen tubes follow a gradient of the attractant, in which case an understanding of the factors that affect this gradient is essential for relating the *in vitro *results to studies of guidance *in vivo*. At longer distances from the source of the attractant, the gradient in our model achieves a steady-state magnitude that approximately varies as the inverse distance to the source: Δ*c *~ (*k*_*p*_/*D*)/*r*. The prefactor *k*_*p*_/*D *reflects the fact that the gradient is essentially maintained by a competition between the ovules releasing the attractant and the attractant diffusing away. The diffusion constant is likely different *in vivo*, and this factor indicates how such a change will affect the range of the attraction. The factor 1/*r *results from the radial symmetry of diffusion on the medium surface, and sets the range at which pollen tubes respond *in vitro*. In going from the planar geometry of the medium *in vitro *to the cylindrical geometry to the funiculus *in vivo *we expect the distance dependence of the concentration to change. In numerical investigations that used a model *in vivo *geometry (unpublished), we found that, at steady-state, the concentration decreased linearly along the axis of the funiculus (i.e., the gradient became constant), and then rapidly decreased on the placental surface. This suggests that the cylindrical geometry of the funiculus may extend the effective range of attraction *in vivo *beyond that in the semi-*in vitro *assay.

At the same time, a signal that provided funicular guidance need not have a very long range on the ovary placenta. Random motility has been shown to provide efficient search strategies in many organisms and would explain the variance in pollen tube growth seen in the ovary chamber [49,50]. Our measured persistence length (~1 mm) is qualitatively consistent with *in vivo *observations that show pollen tubes grow essentially straight for very long distances [49]. The distance over which motion remains correlated is unusually long compared with other cellular systems and comparable to the length of the ovary chamber (2-3 mm long, see [61]). Within the ovary, pollen tubes compete to find an ovule; a long persistence of direction may provide an efficient method to locate an unfertilized ovule for pollen tubes that emerge at the top of the chamber. It would be interesting to determine if a correlation existed between ovary length and pollen tube persistence length in other species of the *Brassicaceae *family.

While it is unclear how the decrease in the rate of growth we observe near the micropyle *in vitro *manifests *in vivo*, the dependence of this decrease on the incubation time suggests that the attractant mediates this decrease, and simulations of our model suggest that it is an important feature of guidance. The decrease in the growth rate could be responsible for the sharp turns observed near the micropyle *in vivo *[22], and the increased turning we observed in our simulations suggests that this is a viable hypothesis. This result does suggest a potentially observable mutant phenotype: elimination of the ability of pollen tubes to decrease their rate of growth would put them at a competitive disadvantage relative to wild-type.

## Methods

### Plant growth and materials

As in [7], pollen was derived from LAT52:GFP transgenic lines in a Columbia background. Stigmas, styles, and ovules were from the *A. thaliana *male sterile mutant, *ms1 *(CS75, Landsberg background). Seeds were sown in soil and stratified at 4°C for 2 days, and plants were grown under fluorescent light (100 *μ*E) for 16 or 24 hrs/day at 40% humidity.

### Semi-*in vitro *pollen guidance assay

Medium was modified slightly from [7] by embedding 1 *μ*m FluoroSphere fluorescent beads that emitted at 540 nm (Invitrogen). Beads were used to correct for drift along the Z-axis in the confocal stacks taken of the system. The presence of the beads did not affect the growth or response of the pollen tubes.

Stigmas, pollen, and ovules were derived from flowers selected at stage 14 [62]. Stigmas were cut at the junction between the style and the ovary using surgical scissors (World Precision Instruments, Sarasota, USA), and were placed horizontally on the pollen growth medium. Stigma were pollinated on the medium with 30-60 pollen grains. Pollen tubes began to emerge two hours after pollination. Ovules were excised from the ovary by first removing the ovary wall with the tip of a 27 gauge needle and then excising the ovules by cutting at the base of the funiculus using a Minutien pin (Fine Science Tools, Foster City, CA, USA). The excised ovules were removed from the ovary and deposited on the surface of the medium, where they were then arranged around the cut stigmas. A 001 insect pin mounted in a pin vice (Fine Science Tools) was used for removal and subsequent manipulation of the ovules. The timing of pollinating the stigmas and placing the ovules was varied according to the desired ovule incubation time (Table 1). For 0-hour incubation times, the stigma was pollinated and two hours later the ovules were placed. For 2-hour incubation times, the ovules were placed, and the stigmas were then immediately pollinated. For 4-hour incubation times, the stigmas were pollinated two hours after the ovules were placed.

### Microscopy

Time-lapse images of GFP-labeled pollen were acquired using an Olympus Fluoview 1000 scanning confocal microscope. Positions of the ovules and stigma were determined using autofluorescence observed with a Cy5.5 filter.

### Correcting stack alignment

The total fluorescence measured at 540 nm in each optical section was used to detect the surface of the medium. Each slice *Z *in the stack had a measured total fluorescence *I*(*Z*), and we normalized this with the maximum fluorescence in the stack . The difference between adjacent intensities  showed a large, consistent peak at the surface of the medium. We defined a peak-peak difference function between two stacks *j *and *k*: . Each stack *j *in the time-lapse was aligned to the initial stack (*k *= 0) by finding the integer value of Δ*Z *that minimized *Q*_*j*0_(Δ*Z*).

### Analysis of images

Pollen tube trajectories were constructed by using ImageJ image analysis software http://rsb.info.nih.gov/ij/download.html. Kalman filtering, as implemented by the Kalman Stack Filter plugin to ImageJ by Chris Mauer, was applied to the stacks before image analysis http://rsb.info.nih.gov/ij/plugins/kalman.html. The tips of the pollen tubes were identified manually. The micropyle of each functional ovule was located by the point where a pollen tube had entered the micropyle, penetrating the ovule. This penetration was assessed with two conditions: pollen tubes had to both reach the micropyle, and subsequent growth had to occur within the focal planes of the ovule autofluorescence. The micropyles of heat-treated ovules were taken to be at the location of the cleft where the funiculus joins the outer integument of the ovule.

Except for the *f*_*closer *_and *f*_*farther *_frequencies, we only included data from pollen tubes growing toward an ovule that was eventually, but not yet, penetrated by a pollen tube to ensure that our conclusions were based on data for guidance toward functional, unfertilized ovules. This restriction was not possible for the heat-treated control because the ovules were never penetrated in that case. The heat-treated control, in these cases, allowed a comparison of growth of pollen tubes between ovules that were capable of attracting the tubes and objects (heated-treated ovules) that were not. This provided a view of how random, or unguided, growth would appear in these measurements.

The angles Δ*θ*, *θ*_*mp*_, and *θ*_*tip *_were calculated for turns in the plane perpendicular to the Z-axis of the confocal stacks, effectively projecting the confocal slices onto a single plane. Distances were confined to this plane to maintain consistency. Values of Δ*θ *were calculated as follows (see Fig. 2C). In each single tube, we subtracted the tip positions at each pair of adjacent time-points *t *and *t*+Δ*t *to give a vector of the growth direction **v**_*tip*_(*t*) = **r**(*t *+ Δ*t*) - **r**(*t*). We the subtracted the position **r**(*t*) from the position of the closest micropyle **r**_*ov *_to form a vector **v**_*ov*_(*t*) = **r**(*t*) - **r**_*ov*_. We calculated the angle between these vectors to yield a value Δ*θ*(*t*) for each time-point *t *in a single tube path.

Values of *θ*_*mp *_and *θ*_*tip *_were calculated using three positions (at *t *- Δ*t*, *t*, and *t *+ Δ*t*) as follows. We calculated the vector of the current growth direction: **v**_*cur*_(*t*) = **r**(*t*) - **r**(*t *- Δ*t*). The new growth direction was the calculated similarly: **v**_*new*_(*t*) = **r**(*t *+ Δ*t*) - **r**(*t*). The direction to the micropyle **v**_*ov*_(*t*) was calculated as in Δ*θ*. For each point, the angle between **v**_*cur*_(t) and **v**_*new*_(t) was denoted *θ*_*tip*_, and the angle between **v**_*cur*_(*t*) and **v**_*ov*_(*t*) was denoted *θ*_*mp*_.

### Descriptive statistics of angular data

Normally the standard deviation of a sample provides a concise summary of the spread of a unimodal distribution. However, because Δ*θ *is an angle, we cannot use linear statistics to describe it. To understand this issue, consider a sample of two angles, 1° and 359°. The linear mean of these angles is (1°+359°)/2 = 180° and the linear standard deviation is 253.1°. However the actual mean direction of these angles is 0°, and the correct circular standard deviation is 1.4°. Because angles are periodic, 359° corresponds to -1°. A correct statistical description of a sample of angles is given by circular statistics [26,27], in which angles are mapped to unit vectors on a circle. This transformation allows the correct calculation of the mean direction and gives a natural circular equivalent to the linear standard deviation; we describe it briefly.

Each angle Δ*θ*_*i *_from a sample of angles is equivalent to a vector of length unity on a circle: . The direction ⟨Δ*θ*⟩ of the angles is found by calculating the mean vector ⟨**u**⟩:

The mean direction ⟨Δ*θ*⟩ is apparent when ⟨**u**⟩ is expressed in polar coordinates:

In this expression ⟨Δ*θ*⟩ is the direction of the mean vector ⟨**u**⟩ and thus the mean direction of the is population of angles {Δ*θ*_*i*_}. The length of ⟨**u**⟩ is *R*, which gives a measure of the spread of the vectors around the circle. The sample circular standard deviation is related to *R *by . This form for σ_0 _is chosen to correspond to the standard deviation of a normal distribution whose tails have been wrapped around a circle [26], and our intuition for Gaussian distributions can be similarly applied to σ_0_: values of σ_0 _≈ 0 indicate a very narrow distribution, while values of σ_0 _→ ∞ indicate an essentially uniform distribution of directions around the circle. To see this, consider *N *angles chosen from a distribution with a very narrow spread around Δ*θ *= 0, and *M *angles chosen from a distribution that is uniform around the circle. In the narrow distribution, the unit vectors **u**_*i *_will be almost identical, their sum will be a vector with length close to *N*, and the length of the mean venctor ⟨**u**⟩ will be close to *R *≈ 1, so *σ*_0 _≈ 0. In the uniform distribution case, the vectors will be uniformly scattered so their directions will essentially cancle; the mean vector will be ⟨**u**⟩ ≈ 0, with length *R *≈ 0, and *σ*_0 _will diverge (*σ*_0 _→ ∞).

### Standard errors, confidence intervals, and tests for statistical significance

Standard errors for *f*_*closer *_and *f*_*farther *_frequencies were calculated by treating each as an estimate of a Bernoulli trial probability, the standard error of which is [63]. Significant differences between these frequencies were determined by *χ*^2 ^testing [63], implemented in the R analysis package [64]. Differences between the frequencies *f*_*farther *_and *f*_*closer *_were initially tested with a 2 × 4 table of dichotomous outcomes: *p *< 0.01 for *f*_*farther *_at all distances and for distances of 50-100 *μ*m. Differences in  were initially tested with a 2 × 3 table: *p *< 0.01 for distances of 0-100 *μ*m. Significance of the pairwise comparisons was tested with a 2 × 2 table.

Standard errors on the circular mean and circular standard deviation of the Δ*θ *angle were calculated using a bootstrap method with 1000 resamples for each statistic [65]. We used a one-sided computational permutation test of the statistic log(*σ*_0_[1]/*σ*_0_[2]), where *σ*_0_[1] and *σ*_0_[2] were the resampled circular standard deviations, with 10,000 different permutations to test for significantly different circular standard deviations. Bootstrap calculations and permutation tests were performed in the R analysis package [64-67]. The confidence interval in the linear model describing persistence was calculated from 10,000 samples generated using the Monte Carlo method included in the program pro Fit [68].

Standard errors in the *θ*_*tip *_angles were calculated by propagating the error in measuring the positions of each tip of the tube. Using the standard propagation of uncertainty, the standard error in the angle *θ *between two lines of length *l*_1 _and *l*_2 _is given by: (*σ*_*θ*_)^2 ^= (*σ*_1_/*l*_1_)^2 ^+ (*σ*_2_/*l*_2_)^2^. Based on the size of the boxes used to track the tips, we assumed an isotropic standard error of 2 pixels (4 *μ*m) for the position of the tips of the pollen tubes.

Standard error on the mean growth rate was estimated as , where *SD *is the maximum likelihood estimate of the standard deviation. The significance of pairwise comparisons of growth rates was determined using Tukey's honest significant difference with a 95% family-wide confidence interval.

### Model of directed turning

Our model for pollen tube response uses the well-studied Langevin equation [69] which separates turning into directed and random components:

The first term describes turning that is proportional to the difference in the fraction of receptors at steady-state bound by the attractant at each side of the tip (Fig. 3A), and the second term adds a random variation to the receptor-mediated response. Here *κ *is the proportionality constant for turning, *c *is the concentration of attractant in units of units of *K*_*D *_(*C *= *c K*_*D*_), Δ*c *is the change in concentration across the tip of the pollen tube, *ξ*(*t*) is a random process that is uncorrelated in time and is Gaussian-distributed with unit variance, and *σ *is the magnitude of the noise. Initial fits of the model to our data revealed no saturation in the turning response, allowing us to simplify the first term. Our model for the receptor response then reduces to Eq. 1.

### Model for the ovule-secreted attractant

To propose a model for the concentration of the attractant, we proceeded similarly to previous models that have studied stable gradients [45-47]. We described diffusion of the attractant on the medium with Fick's law and its release from the ovule with a constant source have radial (rate *k*_*p*_) at the ovule micropyle. The concentration *c*(*r*, *t*) and gradient *G*_*tip *_= *dc*(*r*, *t*)/*dr *have radial symmetry, and their solution as a function of the distance from the origin *r *is(3)

Here *D *is the diffusion constant of the attractant and *E*_1_(⋯) is the exponential integral, a well-characterized special function [70,71]. The parameter *r*_0 _is an offset we introduced to account for the distance that the attractant has to diffuse on the surface of the ovule, where the diffusion coefficient may be different, before entering the thin film of liquid pollen-growth medium that coats the top of the solid agar matrix. This offset also corrects for non-physical behavior near the origin, where a finite amount of attractant is deposited into an infinitesimal region, making the concentration there infinite [47].

To determine how experimental errors would affect the model parameters, we used our Gaussian model for the error in tip positions (s. d. of 4 *μ*m) to generated 10,000 synthetic data sets. To estimate confidence intervals for the model parameters, we fit theses data sets. Table 3 reports the 90% range of values for these fits.

### Model of random turning

To access short regions of growth, we took advantage of the fact that, on the length scales of the experiment, pollen tube growth is smooth with few sharp angles and fit the points in the time-lapse with a spline curve to study the growth at intervals as short as 5 *μ*m of growth. The change in direction of a pollen tube was found by finding the angle between the direction of growth at some distance along the tube *s *and a new direction of growth after the tube had grown a distance *δs *(Fig. 5B). We write this as the turning angle *θ*_*tip*_(*s*, *s *+ *δs*). Its cosine, cos *θ*_*tip*_(*s*, *s *+ *δs*), measures the correlation between the vector for the direction of growth at *s *and the vector at *s *+ *δs*: **v**(*s*)·**v**(*s *+ *δs*) = cos*θ*_*tip*_(*s*, *s *+ *δs*), where **v**(*s*) has been normalized to have unit length. Averaging this quantity over all lengths of *δs *provides a measure of the average amount of the original direction retained in the new direction: ⟨**v**(*s*)·**v**(*s *+ *δs*)⟩_*s *_= ⟨(cos*θ*_*tip *_(*s*, *s *+ *δs*)⟩_*s*_, where the subscript *s *indicates an average over all possible lengths *δs*. This correlation function often has an exponential form:

where the last approximation is valid for short amounts of growth relative to the persistence length (*δs *≪ *L*). Graphically plotting ⟨cos*θ*_*tip*_⟩ against *δs *revealed a roughly linear relationship, with an intercept close to unity and a slope just below zero, consistent with a long persistence length (Fig. 5B). Specifically, we fit this relationship with the linear model ⟨cos*θ*_*tip*_(*δs*)⟩ = (1 + *b*) - *δs*/*L*, with *b *= -7.0 × 10^-4 ^and *L *= 1074.02 *μ*m. The parameter *b *is the deviation from the intercept at unity and was not statistically different from *b *= 0. The parameter *L *is the persistence length, and was found to fall in range 1042.70-1108.68 *μ*m with 99.9% confidence. Although the data showed non-random oscillations around the linear fit, the deviations were small (the two largest deviations were a 5% error at *δs *= 115 *μ*m and 6% error at *δs *= 385 *μ*m). The long persistence length *L *indicates that the probability of making a turn *θ*_*tip *_peaks sharply around *θ*_*tip *_= 0, indicating that ⟨cos*θ*_*tip*_⟩ ≈ 1 - ⟨*θ*_*tip*_^2^⟩. and that the distribution of *θ*_*tip *_can be described as a sharply-peaked Gussian with mean 0 and variance . This allows us to set the parameter *σ *which scales the random component of a turn to .

### Fitting parameters

Given the parameters *κ*, *k*_*p*_, *D*, and *r*_0_, our model calculates the mean direction a tube should turn from (a) the location of a pollen tube tip, (b) the direction the tip is growing, (c) the time the ovules have had to release a guidance cue, and (d) the location of the ovules. Our experimental pollen tube trajectories contained both this information and the angle the tube actually turned. We used a *χ*^2 ^metric to evaluate how well a set of parameters described the experimental data:

Here *σ*_*tip *_includes both the error in measuring *θ*_*tip *_and the fluctuations predicted by the *σ *parameter of the model. The term *θ*_*i *_is the predicted mean angle for turning:

where the subscript *i *denotes each separate direction to the micropyle *θ*_*mp*_(*i*), which has position **r**_*i *_and occurs at time *t*_*i*_, and Δ*c*(**r***i*, *θ*_*mp*_(*i*), *t*_*i*_) is the change in concentration across the tip of the pollen tube, which depends on the same quantities. Here the width of the pollen tube, Δ*L*, is absorbed into the constant *κ *with no loss of generality.

Fits obtained using the Levenberg-Marquardt algorithm to minimize *χ*^2 ^[71] often became stuck in local minima. We found that a Powell's level set method [71], implemented in Scientific Python [72,73], proved much more robust. When fitting, the adjustable parameters should be made as independent as possible. The term Δ*c*(**r**, *θ*_*mp*_, *t*) has a prefactor of *k*_*p*_/*D*, which makes the parameters *κ*, *k*_*p*_, and *D *highly covariant. We removed this dependence by introducing a combined parameter *κ*' = *κ*(*k*_*p*_/*D*) and fitting *κ*' and the diffusion constant *D *as two independent parameters (*D *can be left as a separate parameter because of its appearance in the exponential in Eq. 3). Written in terms of the parameters *κ*', *D*, and *r*_0 _the mean response *θ*_*i *_is then

where we write  to emphasize that we removed the *k*_*p*_/*D *prefactor, but that these terms are still parameterized by *D *and *r*_0_. To understand the resulting fits, we assessed the turning response of the model and compared this response at different distances with the experimental responses (Fig. 5A).

### Model for pollen tube growth rates

To model how the growth rate decreased near the micropyle, we assumed that the pollen tubes were responding to higher gradients of the attractant. A simple way to model this is to assume that a pollen tube periodically adjusts its rate of growth based on the difference in the concentration it perceives across its tip: *v*_*new *_= *v*_*min *_+ (*v*_*old *_- *v*_*min*_)/(*k*_*v *_Δ*c *+ 1), where *k*_*v *_modulates the response to the change in concentrations. At low values of Δ*c*, *v*_*new *_≈ *v*_*old*_, while at high values, *v*_*new *_approaches *v*_*min*_. This formulation is consistent with our general observation that once the rate of growth of a pollen tube had slowed, it never substantially increased. Our model is essentially a continuously-sampled formulation of *v*_*new*_:(4)

where *τ *is the timescale for slowing growth in response to a gradient. To fit this model, we used the rate of growth between each time point with *t *= 20 min. We noticed that tubes would grow as slowly as 0.5 *μ*m/min when near the micropyle and accordingly set *v*_*min *_= 0.5 *μ*m/min. The parameters *k*_*v *_and *τ *were fit with the robust fitting method provided by the pro Fit analysis program [68] to obtain values *k*_*v *_= 533.39 (1/dimensionless concentration units) and *τ *= 19.20 min. The model showed good agreement with the average rate of growth until very close to the micropyle (*R *~ 10 *μ*m), where it predicted a higher average rate of growth than observed.

### Simulation protocol

Each simulation has a set of virtual pollen tubes, each of which has an index *j*, a current position **r**_*j*_, as well as a rate and a direction of growth (the magnitude and direction of vector **v**_*j*_). In addition, each simulation also has a list of ovule micropyle locations. We implement the model (Eqs. 1 and 4) by choosing discrete time-steps of length Δ*t *= 0.1 min and using the model equations to evolve the position and direction of growth of each virtual pollen tube tip. Specifically, at each step in time Δ*t*, the simulation iterates through the list of pollen tubes and does the following:

1. If the virtual pollen tube has been previously "captured" by coming within a short distance (10 *μ*m) of the virtual micropyle, or growing more than 800 *μ*m from the center of the simulation, then the simulation ignores it.

2. The virtual tip is advanced based on the previous direction: **r**_*j*_(*t *+ Δ*t*) = **r**_*j*_(*t*) + **v**_*j*_(*t*)Δ*t*

3. The concentration and gradient at position **r**_*j*_(*t *+ Δ*t*) and time *t *+ Δ*t *are calculated:  and  by adding up the contributions of each virtual ovule, given by Eq. 3 and its gradient . Here we use the bar over  to indicate that the refactors of the concentration and gradient terms have been *k*_*p*_/*D *absorbed in the parameter *κ*'.

4. We pick a random number *z*_*j *_from a Gaussian distribution with mean *μ *= 0 and variance *σ*^2 ^= 2*v*/*L *(Section 2.4). The turning angle (*δθ*_*j*_(*t *+ Δ*t*) is calculated from Eq. 1: , where  is the gradient calculated in step 3 and *θ*_*mp *_is the angle between the direction **v**_*j*_(*t*) of the previous step and the gradient .

5. In simulations where the rate of grow changes (denoted S+ in the text), the new rate of growth *v*_*j*_(*t *+ Δ*t*) is calculated according to Eq. 4, where the old rate of growth is of growth *v*_*j*_(*t*) the magnitude of **v**_*j*_(*t*):

6. **v**_*j*_(*t *+ Δ*t*) is generated by rotating **v**_*j*_(*t*) by *δθ*_*j*_(*t *+ Δ*t*) and rescaling the vector to have magnitude *v*(*t*+Δ*t*).

7. If the new position of the virtual pollen tube, **r**_*j*_(*t *+ Δ*t*), is within 10 *μ*m of the ovule icropyle or has grown more than 800 *μ*m from the center of the simulation, then the virtual tube is marked as "captured," and is ignored in subsequent iterations.

These steps are continuously iterated until the simulation ends.

In addition to this algorithm, each simulation requires the location of the virtual ovules and a set of initial positions and directions for the virtual pollen tubes. To enable direct comparison between our simulations and the experimental data, we used the same micropyle locations, initial pollen tube locations, and incubation time as an experimental replicate. Each group of incubation times simulated (0-hr, 2-hr, and 4-hr) used the same replicates as in the experiments. To set the initial pollen tube directions, we used a vector between the first and second experimental positions for each tube, scaled by the time interval between the measurements (20 min). Our simulations were unconstrained by the requirements of image analysis, which meant that we could run an unlimited number of pollen tubes in each virtual replicate, and we chose to use 500 tubes in each replicate to increase the statistics of each run. Each experimental replicate had 20-40 tubes, which gave us 20-40 initial conditions (positions and directions). We randomly, and uniformly, chose one of these initial conditions for each of our virtual pollen tubes, essentially treating the experimental set of initial conditions as a bootstrap distribution. Because of the random variations in the turning angle (the random number in step 4), two virtual tubes that start with the same initial condition will ultimately have distinct paths. This mirrors the experimental behavior where many of the tubes initially emerged from the transmitting tract in a tightly packed formation, growing in the same direction, but then the tubes would grow randomly on the medium, spreading out to a fan-like distribution.

## Authors' contributions

SFS performed the experiments and analyzed the data described in this study. SFS, MJR, DP, and ARD conceived, designed, coordinated this study and drafted the manuscript. PB and MT contributed simulation and analysis tools to this study. All authors read and approved the final manuscript.
